# Protective Role of Sulforaphane against Physiological Toxicity of Triphenyltin in Common Carp (*Cyprinus carpio haematopterus*)

**DOI:** 10.3390/antiox13101173

**Published:** 2024-09-26

**Authors:** Bingke Wang, Chunnuan Zhang, Jianshuang Ma, Yanhui Wang, Ling Zhang, Xingli Yang, Tao Jia, Kaisong Zhang, Qin Zhang

**Affiliations:** 1 Henan Academy of Fishery Sciences, Zhengzhou 450044, China; bkwang1992@163.com (B.W.);; 2 Henan Fishery Engineering Technology Research Center, Zhengzhou 450044, China; 3 College of Animal Science and Technology, Henan University of Science and Technology, Luoyang 471003, China

**Keywords:** sulforaphane, triphenyltin, physiological toxicity, growth performance, immunity, antioxidant

## Abstract

This experiment mainly explored the protective role of sulforaphane (SFN) against physiological toxicity of triphenyltin (TPT) in *Cyprinus carpio haematopterus*. In total, 320 Fish (56.90 ± 0.40 g) were randomly divided into four groups with four replicates each. The control group was fed the basal diet, the TPT group (TPT) was exposed to 10 ng L^−1^ TPT on the basis of the control group, the SFN + TPT group (TPT + SFN) was fed a diet supplemented with 10 mg kg^−1^ SFN on the TPT group, and the SFN group (SFN) was fed a diet supplemented with 10 mg kg^−1^ SFN. After 56 days of breeding trials, the results showed that TPT exposure resulted in a remarkable decrease (*p* < 0.05) in final weight, weight gain rate (WGR), specific growth rate (SGR), and condition factor (CF), but an increase (*p* < 0.05) in feed conversion ratio (FCR) and hepatosomatic index (HSI) of fish. TPT treatment decreased (*p* < 0.05) the amounts of hematocrit (Hct) and hemoglobin (Hb), plasma complement component 3 (C3) and C4 contents, alternative complement pathway (ACH50), acid phosphatase (ACP) and lysozyme (LZM) activities, liver glutathione (GSH) content, catalase (CAT), superoxide dismutase (SOD), glutathione peroxidase (GPX) activities, interleukin 10 (*IL-10*), and *SOD* mRNA expressions, but increased (*p* < 0.05) plasma alanine aminotransferase (ALT) and aspartate aminotransferase (AST) activities, liver malonaldehyde (MDA) content, tumor Cyclooxygenase 2 (*COX2*), and necrosis factor α (*TNFα*), *IL-1β*, and *MDA* mRNA expressions. A histological analysis of the liver showed that a higher occurrence rates of the hepatocyte hypertrophy, nuclear disappearance and hepatocyte vacuolization was observed in the hepatocytes of fish exposed to TPT, and it was accompanied by the dilation of hepatic sinusoids. In addition, the toxicity induced by TPT was significantly improved in the groups that were treated with SFN, and SFN was able to improve growth performance and immunity, alleviate TPT-induced changes in inflammatory factors, ameliorate oxidative stress, and increase the activity of antioxidant enzymes (*p* < 0.05). The addition of SFN also alleviated liver damage caused by TPT and protected the structural integrity of the liver. Overall, these findings suggest that TPT inhibited the growth, immunity, and antioxidant capacity of *Cyprinus carpio haematopteru.* Dietary SFN could be beneficial for growth promotion, immunity, antioxidant capacity, and protection of liver structural integrity. Therefore, SFN is a prospective feed supplement for ameliorating the damage caused to fish by TPT contamination.

## 1. Introduction

Organotin compound has been widely used as an agricultural pesticide, fungicide, plastic stabilizer, wood preservative, anti-fouling paint, and other products since the 1960s [1]. TPT and Tributyltin (TBT) are the major organotin compounds and are ubiquitous in the aquatic environment due to their widespread use. Most previous studies have focused on TBT [2,3], while little research had been conducted on TPT. With the increasing concern about the safety of TPT, TPT has gradually come into people’s field of vision. High concentrations of TPT have been reported to be detected in the coastal, Yangtze River, Jialing River, and Three Gorges Reservoir regions in China [4]. In fact, TPT has a bioaccumulation effect, as it can enter and gradually accumulate along the food chain, eventually leading to higher concentrations of TPT in top predators (e.g., humans), and the ecotoxicological assessments of TPT suggest that aquatic organisms might be confronted with serious risks due to long-term exposure to TPT [5].

Numerous studies have shown that TPT exposure has adverse effects on fish growth. Japanese medaka (*Oryzia lapites*) has been reported to be remarkably affected by TPT; 1.0 μg L^−1^ TPT could reduce the growth and fertility of Japanese medaka [6]. Environmental-related concentrations of TPT not only inhibited the growth of larval coral reef fish (*Amphiprion ocellaris*), but also disrupted their pigmentation and skeletal development [7]. There is also research to prove that TPT exposure could induce abnormal development in zebrafish larvae (*Danio rerio*) and inhibit growth by interfering with the hypothalamus-pituitary–thyroid axis [8]. Furthermore, several studies have demonstrated that TPT could cause immunosuppression and oxidative stress in fish. TPT has been found to have oxidative stress and immunosuppressive effects on goldfish (*Carassius auratus*) [9]. TPT may interfere with the activities of antioxidant enzymes (e.g., CAT, SOD, and glutathione S-transferases) [10]. TPT disrupted lipid metabolism in common carp (*Cyprinus carpio*), while combined exposure to TPT and Microplastics led to an immunosuppressive response [11]. A trial also evaluated the co-occurrence risk of norfloxacin and TPT in the environment on aquatic organisms and showed that their co-exposure enhanced immunosuppressive functions and metabolic disorders in carp (*Cyprinus carpio*) [12]. In addition, experimental studies also suggested that TPT could change the composition of intestinal microorganisms in marine medaka (*Oryzias melastigma*) [1], interfere with androgen synthesis in rainbow trout, and cause damage to the thyroid gland [13]. There are many studies on TPT, but most of them focus on its impact in aquatic organisms and the environment, with little mention of countermeasures and solutions. How to deal with the various problems caused by TPT will be the focus of our research and direction.

Recently, due to their immunomodulatory and antioxidant effects, plant extracts have offered an alternative to antibiotics in aquaculture [14]. Sulforaphane (SFN), a naturally occurring isothiocyanate compound, is derived from vegetables in the genus Brassica, Eruca, and Raphanus [15]. SFN exhibits remarkable immune-enhancing activity and is an excellent immunoprotected agent, enhancing the human body’s overall immune functions [16]. SFN has also been shown to have beneficial antioxidant and anti-inflammatory effects through action on the Keap1/Nrf2/ARE pathway [17]. Previous studies have demonstrated that SFN significantly decreases oxidative damage and improves the antioxidant capacity of *Litopenaeus vannamei* [15]. Our previous study also showed that SFN could promote the growth of *Cyprinus carpio haematopterus*, enhancing its antioxidant and immunity capacity [18]. Thus, SFN is a potential additive against oxidative stress, but it remains unclear whether SFN has a protective role against physiological toxicity of TPT in *Cyprinus carpio haematopterus*. More studies are needed to verify the physiological toxicity of TPT and the protective effect of SFN against it.

Common carp (*Cyprinus carpio haematopterus*) is widely farmed in China and is also frequently used for toxicological experiments [12]. As a natural plant extract, SFN has great potential in the development of immune-enhancing feeds for fish. Therefore, the objective of this experiment was mainly to explore the environmental health risks posed by organotin pollutants through the TPT environmental exposure experiment and protective role of SFN against physiological toxicity of TPT in *Cyprinus carpio haematopterus.*

## 2. Materials and Methods

### 2.1. Chemicals and Experimental Diets

The TPT used in this experiment was purchased from Sigma Aldrich (98%, CAS:1124-19-2). It was dissolved in absolute ethanol to a concentration of 10 μg mL^−1^ (33.1 nmol mL^−1^). Then, add the solution directly to the exposed water. The concentration of TPT was designated according to Zhang et al. [10]. SFN was purchased from Chengdu Must Biotechnology Co., Ltd. (Chengdu, China) (CAS: 4478-93-7), boasting a purity of 98% or higher.

The composition of the experimental diets was shown in Table 1. The ingredients were mixed with oil and then mixed well with an appropriate amount of water. Then, pellets were made into pellet diets using a pelletizer and dried in a cool place. In total, 10 mg kg^−1^ of SFN was added to the basal diets; the concentration of SFN was designated according to our previous study [18]. Once dried, the feed was crushed and sieved to the appropriate particle sizes. Stored at 20 °C until use.

### 2.2. Fish and Experimental Design

*Cyprinus carpio haematopterus* were taken from an aquaculture farm in Luoyang (Luoyang, China), and the experiment was carried out in t in a professional aquaculture laboratory. The basal diet was fed for two weeks prior to the feeding trial. After acclimatization, 320 fish (56.90 ± 0.40 g) were randomly allocated to 16 tanks (0.8 m × 0.6 m × 0.4 m, L:W:H) with 20 fish in each tank. The fish are divided into four groups with four repeats: the control group, the 10 ng L^−1^ TPT group, the 10 ng L^−1^ TPT + 10 mg kg^−1^ SFN group, and the 10 mg kg^−1^ SFN group. The fish were hand-fed three times a day (07:30, 12:00, and 16:30) for eight weeks until apparently satiated. During the experiment period, the fish were maintained under the conditions as follows: temperature of the water was between 25 and 28 °C; pH varied between 7.1 and 7.3; the dissolved oxygen (DO) was kept at 6.0 mg L^−1^; and total ammonia nitrogen remained below 0.04 mg L^−1^. To ensure a consistent effective concentration of TPT during exposure, 25% of the water in each tank was changed daily, with immediate re-administration of the treatment following renewal.

### 2.3. Sample Collection

After the feeding trials, the fish were hungry for 24 h prior to sampling to empty their digestive contents. Count and weigh all the fish in each fish tank. Subsequently, 4 fish were randomly selected from each tank (16 fish per group) and we anesthetized the fish with an anesthetic. Following this, blood was drawn from the tail vein using a syringe containing sodium heparin, and was split into two parts. The first portion was centrifuged at 3000× *g* for 10 min at 4 °C, and the plasma (for innate immune parameter assays) was stored at −80 °C until the trial. The second portion was transferred to a heparin-coated Eppendorf tube for hematological parameter analysis. Subsequently, visceral and liver samples were collected from the fish, weighed, and used to calculate biometric parameters. A portion of the liver tissue was taken for histological analysis, with the remainder stored in liquid nitrogen for future analysis.

### 2.4. Analytic Procedures

#### 2.4.1. Hematological and Plasma Biochemical Parameters

A Thomas blood cell counter and Darcy’s dilution were used to count the red blood cells (RBCs). The concentration of hemoglobin (Hb) was determined using the cyanmethemoglobin method. The hematocrit (Hct) percentage was measured using microhematocrit capillary tubes [19]. Plasma alternative complement pathway (ACH50) activity and complement component 3 (C3) and C4 levels were determined according to the methods of Montero et al. [20] and Tang et al. [21]. Measurement of plasma acid phosphatase (ACP) activity was carried out using the phenylphosphate method [22]. Plasma lysozyme (LZM) activity was measured using the turbidity method [23]. Analysis of plasma alanine aminotransferase (ALT) and aspartate aminotransferase (AST) activities was performed using the method described by Krajnović-Ozretić and Ozretić [24].

#### 2.4.2. Histological Examination

Liver tissue samples preserved in formaldehyde solution were dehydrated step by step, embedded in paraffin, and made into 4 μm sections. They were then stained with hematoxylin and eosin staining (H&E). Finally, they were photographed with a microscope.

#### 2.4.3. Analysis of Antioxidant Status

Liver catalase (CAT) activity was quantified employing a commercially available kit in accordance with the approach delineated by Goth [25]. Determination of liver malondialdehyde (MDA) content and superoxide dismutase (SOD) activity was performed using the barbituric acid reaction chronometry and xanthine oxidase methods [26,27], respectively. The activity of glutathione peroxidase (GPX) and the concentration of glutathione (GSH) in the liver were determined in accordance with Lygren et al. [28].

#### 2.4.4. Related Gene Expression Analysis

Total RNA was extracted from the liver using Trizol Reagent RNA kit, RNA concentration was measured, reverse transcription was performed according to the instructions, and cDNA was obtained for PCR amplification. ROX (0.2 μL), primers (10 μmol L^−1^, 0.2 μL), cDNA (2 μL), 10 μL of SYBR Green (10 μL), and sterile water (7.6 μL) were added successively to a 96-microwell plate to make a total of 20 μL. The primer sequences are shown in Table 2 and were designed using Primer 5.0 software, following the instructions for the SYBR^®^ Premix Ex Taq^TM^ II kit from Takara and adding samples to the machine for amplification. Normalization of gene expression was performed with the 2^−ΔΔCt^ method [29].

#### 2.4.5. Calculation Formula

The growth parameters adopted in this study were calculated as follows:

(1) $$\mathrm{Weight}\;\mathrm{gain}\;\mathrm{rate}\;(\mathrm{WGR},\;\%)=\frac{W_{f}-W_{i}}{W_{i}}\times 100$$

(2) $$\mathrm{Specific}\;\mathrm{growth}\;\mathrm{rate}\;(\mathrm{SGR},\;\%\;\mathrm{day}{-}^{1})=\frac{{LnW}_{f}-{LnW}_{i}}{T}\times 100$$

(3) $$\mathrm{Feed}\;\mathrm{conversion}\;\mathrm{ratio}\;(\mathrm{FCR})=\frac{total\;feed\;intake}{total\;body\;weightgain}$$

(4) $$\mathrm{Condition}\;\mathrm{factor}\;(\mathrm{CF},\;\%)=\frac{body\;weight}{{body\;length}^{3}}\times 100$$

(5) $$\mathrm{Hepatosomatic}\;\mathrm{index}\;(\mathrm{HIS},\;\%)=\frac{liver\;weight}{body\;weight}\times 100$$

(6) $$\mathrm{Viscera}\;\mathrm{index}\;(\mathrm{VSI},\;\%)=\frac{viscera\;weight}{body\;weight}\times 100$$

W_i_ is the initial weight, W_f_ is the final weight, T is days of experiment.

### 2.5. Statistical Analysis

The experiment was randomly divided into four groups (sixteen tanks in total) and four tanks per group (four parallel). Four fish were randomly selected from each tank, and there were sixteen data for each treatment group. Some data that were far away from the others were excluded before analyzing. Using SPSS 22 software to analyze all the data. Dietary TPT, SFN, and their interactions were analyzed using two-way ANOVA. If there was a significant difference found between the factors (*p* < 0.05), the differences between the groups were analyzed using one-way ANOVA. All analysis results were reported in the form of means ± standard error of the mean (SEM).

## 3. Results

### 3.1. Effects of TPT and SFN on the Growth Performance of Cyprinus carpio haematopterus

Growth performance of *Cyprinus carpio haematopterus* were presented in Table 3. Fish that were exposed in TPT showed significant decreases (*p* < 0.05) in final weight, WGR, SGR, and CF, along with significant increases (*p* < 0.05) in FCR and HSI compared to those of the control fish. However, SFN supplementation significantly increased (*p* < 0.05) final weight, WGR, SGR, and CF along with significant decreasing (*p* < 0.05) FCR compared to those in the TPT group. The application of SFN alone showed no significant differences (*p* > 0.05) compared to the control groups. No difference (*p* > 0.05) was observed in VSI among all of the treatments. In addition, final weight, WGR, SGR, and HSI were all significantly (*p* < 0.05) affected by the interaction between TPT and dietary SFN.

### 3.2. Effects of TPT and SFN on the Hematological and Plasma Immune Parameters of Cyprinus carpio haematopterus

**Figure 1:**
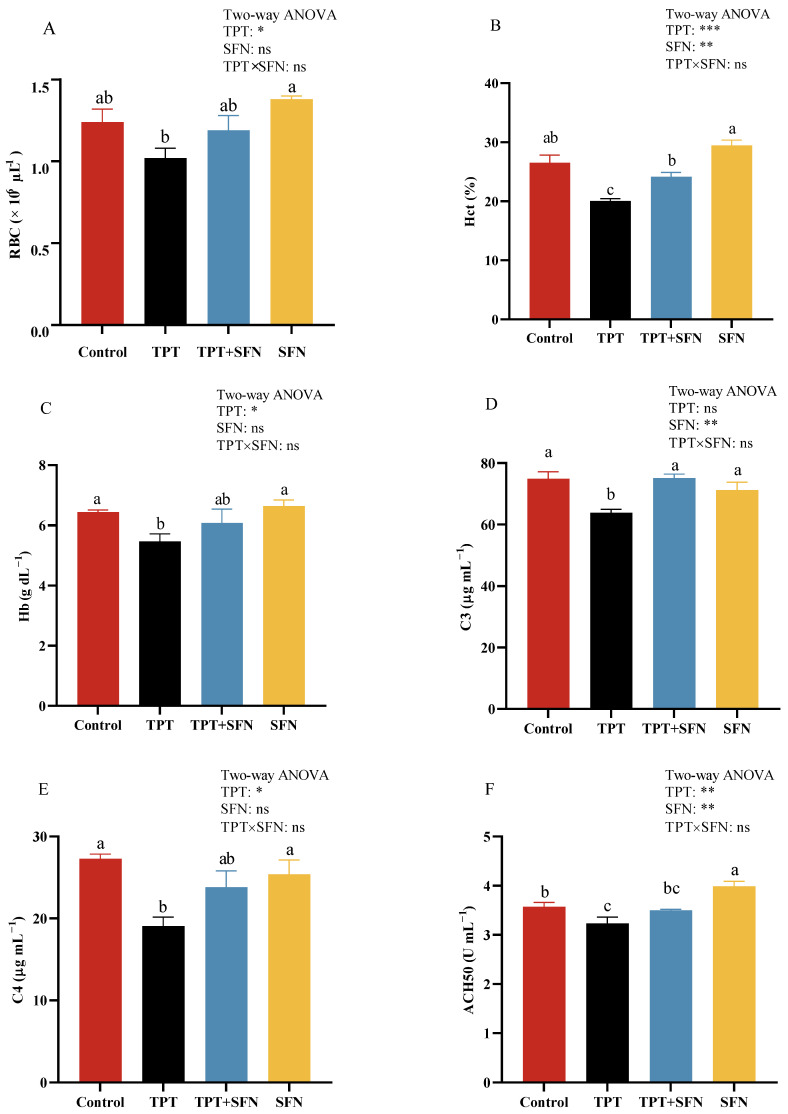

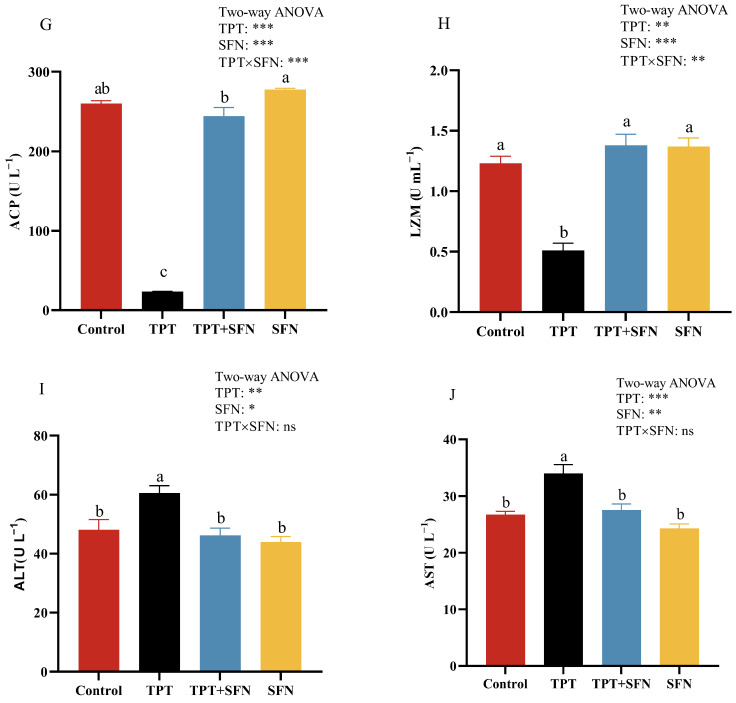
(**A**–**J**) Effects of TPT and SFN on the hematological and plasma immune parameters of *Cyprinus carpio haematopterus*. RBC, red blood cell; Hct, hematocrit; Hb, hemoglobin; C3, complement component 3; C4, complement component 4; ACH50, alternative complement pathway; ACP, acid phosphatase; LZM, lysozyme; AST, aspartate aminotransferase; ALT, alanine aminotransferase. * *p* < 0.05, ** *p* < 0.01, *** *p* < 0.001, ns, not significant. Values are expressed as means ± SEM. Bars assigned with different letters are significantly different (*p* < 0.05).

As can be seen from Figure 1, the amounts of Hct and Hb, plasma C3 and C4, contents as well as ACH50, ACP, and LZM activities were significantly decreased (*p* < 0.05) in fish by TPT exposure compared with the control group, while plasma ALT and AST activities were notably higher (*p* < 0.05). However, treatment with SFN prevented a significant decrease in Hct amounts, plasma C3 content, as well as ACP and LZM activities that were induced by TPT exposure, but showed a decrease (*p* < 0.05) in plasma ALT and AST activities. Compared with the control group, plasma ACH50 activity was significantly higher in the SFN group (*p* < 0.05). Furthermore, there was an interaction between TPT and dietary SFN on plasma ACP and LZM activities (*p* < 0.05).

### 3.3. Effects of TPT and SFN on Liver Immune-Related Gene Expressions of Cyprinus carpio haematopterus

**Figure 2:**
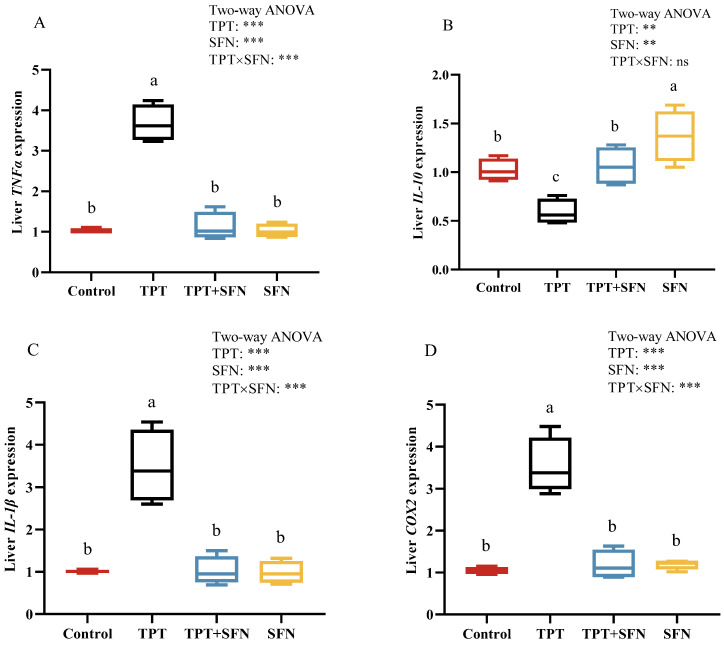
(**A**–**D**) Effects of TPT and SFN on liver immune related gene expressions of *Cyprinus carpio haematopterus*. *TNF-α*, tumor necrosis factor α; *IL-10*, interleukin 10; *IL-1β*, interleukin-1β; *COX2*, Cyclooxygenase 2. ** *p* < 0.01, *** *p* < 0.001, ns, not significant. Values are expressed means ± SEM. Bars assigned with different letters are significantly different (*p* < 0.05).

As seen in Figure 2, the mRNA expressions of liver *IL-1β*, *TNFα*, and *COX2* were significantly increased (*p* < 0.05), while *IL-10* mRNA expression decreased significantly (*p* < 0.05) in TPT-exposed fish compared to control. However, the addition of SFN significantly inhibited (*p* < 0.05) the mRNA expressions of *IL-1β*, *TNFα*, and *COX2* and promoted the expression of *IL-10* mRNA compared to the TPT-treated group. There was a significantly higher liver *IL-10* mRNA expression in the SFN group than in the control group (*p* < 0.05). In addition, there was an interaction between TPT and dietary SFN on liver *TNFα*, *IL-1β*, and *COX2* mRNA expressions (*p* < 0.05).

### 3.4. Liver Histological Examinations of Cyprinus carpio haematopterus

**Figure 3:**
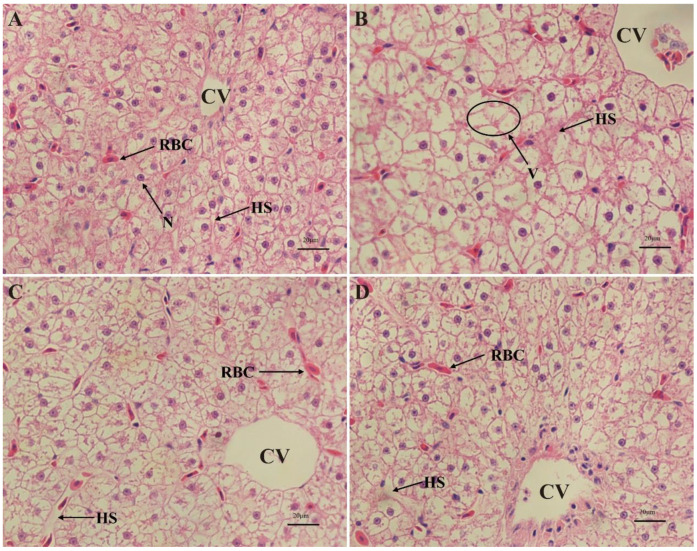
(**A**–**D**) Photomicrographs of liver tissue of *Cyprinus carpio haematopterus* (H&E staining, scale bars = 20 μm). (**A**), control group; (**B**), TPT group; (**C**), TPT + SFN group; (**D**), SFN group. CV, central vein; HS, hepatic sinusoid; N, nucleus; RBC, red blood cell; V, vacuolation.

Liver histological examinations of *Cyprinus carpio haematopterus* were shown in Figure 3. No histopathological change was identified in the liver of the control group. The structure of the liver plate was clear and orderly arranged. The cytoplasm of the liver cell was uniform. The nucleus was regular and round, and it was located in the center of the cell. Hepatic sinusoids were morphologically normal and distributed between liver cells (Figure 3A). A higher occurrence rates of the hepatocyte hypertrophy, nuclear disappearance, and hepatocyte vacuolization was observed in the hepatocytes of fish exposed to TPT, and it was accompanied by the dilation of hepatic sinusoids (Figure 3B). However, liver damage caused by TPT was alleviated with the addition of SFN compared to the TPT group (Figure 3C). In addition, there were no obvious abnormalities in the basic structure of the liver in the SFN group (Figure 3D).

### 3.5. Effects of TPT and SFN on Liver Antioxidant Enzyme Activities of Cyprinus carpio haematopterus

**Figure 4:**
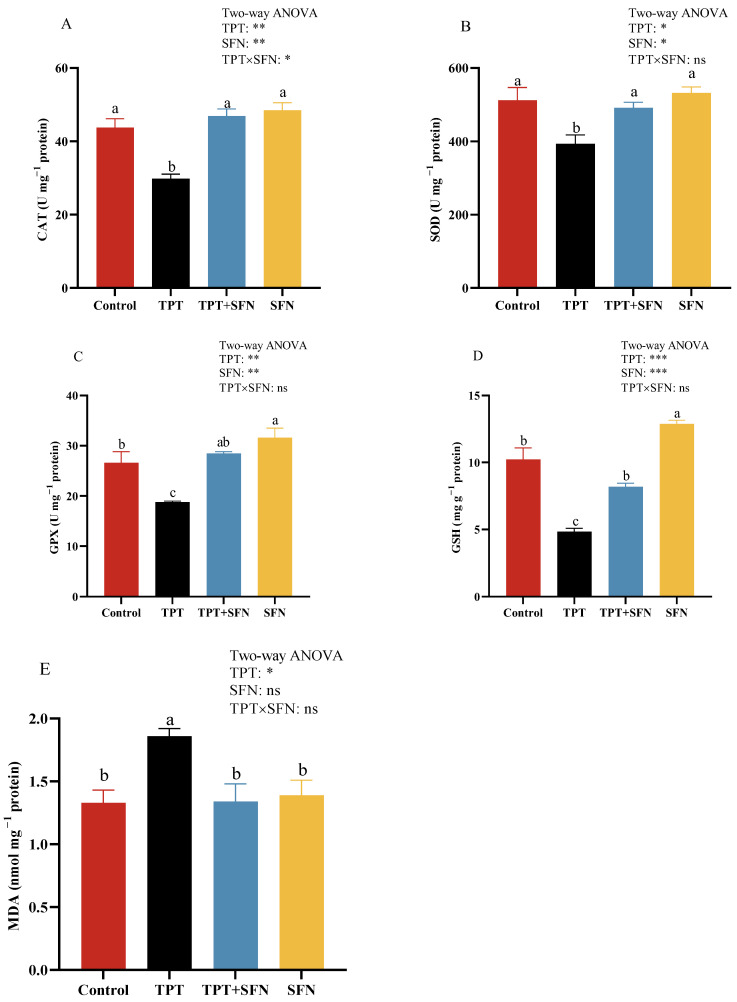
(**A**–**E**) Effects of TPT and SFN on liver antioxidant enzyme activities of *Cyprinus carpio haematopterus*. CAT, catalase; SOD, superoxide dismutase; GPX, glutathione peroxidase; GSH, glutathione; MDA, malonaldehyde. * *p* < 0.05, ** *p* < 0.01, *** *p* < 0.001, ns, not significant. Values are expressed means ± SEM. Bars assigned with different letters are significantly different (*p* < 0.05).

Liver antioxidant capabilities of *Cyprinus carpio haematopterus* were shown in Figure 4. Fish in TPT group showed significant decreases (*p* < 0.05) in liver CAT, SOD, GPX activities, and GSH content, along with significant increases (*p* < 0.05) in MDA content compared to the control fish. However, SFN supplementation significantly increased (*p* < 0.05) CAT, SOD, GPX activities, and GSH content, along with significant decreasing (*p* < 0.05) MDA content compared to those of the TPT group. The SFN group showed significant elevations (*p* < 0.05) in liver GPX activity and GSH content compared to the control group. Furthermore, liver CAT activity was significantly affected (*p* < 0.05) by the interaction between TPT and dietary SFN.

### 3.6. Effects of TPT and SFN on Liver Antioxidant Related Gene Expressions of Cyprinus carpio haematopterus

**Figure 5:**
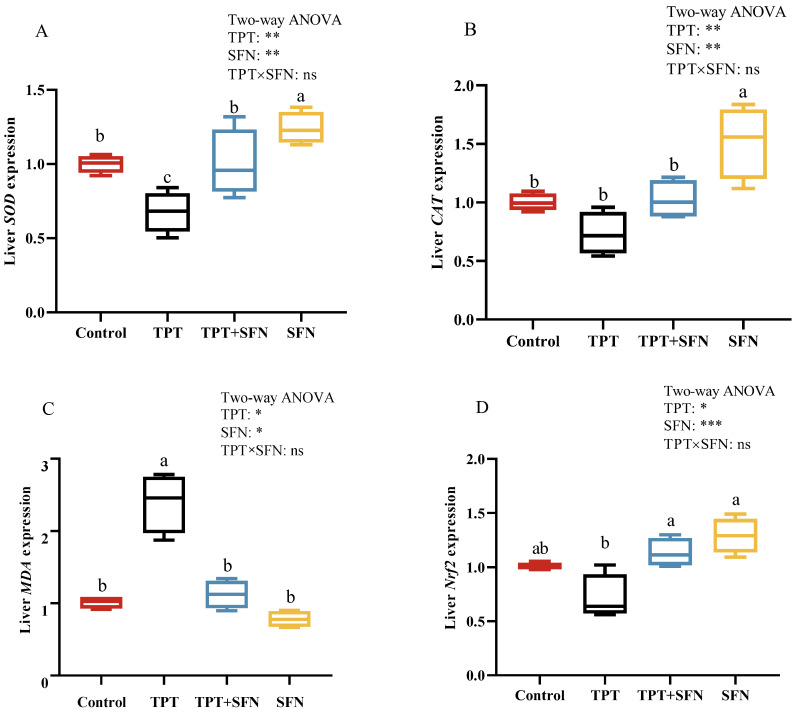
(**A**–**D**) Effects of TPT and SFN on liver antioxidant-related gene expressions of *Cyprinus carpio haematopterus*. *Nrf2*, nuclear factor E2 related factor 2. * *p* < 0.05, ** *p* < 0.01, *** *p* < 0.001, ns, not significant. Values are expressed means ± SEM. Bars assigned with different letters are significantly different (*p* < 0.05).

As presented in Figure 5. A significant decrease (*p* < 0.05) in *SOD* mRNA expression and a significant increase (*p* < 0.05) in *MDA* expression were observed in the livers of fish exposed to TPT compared to the control group. However, *SOD* and *Nrf2* mRNA expressions were significantly higher and *MDA* mRNA expression was significantly lower after SFN application compared to the TPT group. The application of SFN alone presented significantly higher in *SOD* and *CAT* mRNA expressions compared to those in the control fish.

## 4. Discussion

In the present study, TPT exposure resulted in a remarkable decrease in final weight, WGR, SGR, and CF, but an increase in FCR and HSI of *Cyprinus carpio haematopterus*. This was not surprising, since TPT exposure could inhibit skeleton development and impair mobility and feeding capabilities [7]. This might inevitably result in slow growth and feed waste, with consequent decreased final weight, WGR, SGR, and CF, but increased FCR. The increase in HSI might be attributed to pathological hyperplasia of the liver, which grew larger to secrete more digestive fluid and digest feed better. This result was in accordance with previous studies on salamanders (*Ambystoma barbourin*) [30]. In addition, in contrast to TPT exposure alone, the supplementation of SFN in diets resulted in a remarkable increase in final weight, WGR, SGR, and CF, but a decrease in FCR. According to our previous studies, SFN could effectively improve growth performance parameters of *Cyprinus carpio haematopterus* [18]; this might be attributed to the effect of SFN on intestinal digestive enzyme activity and microbiota, and immune and antioxidant ability. Further in-depth studies were warranted to elucidate this.

Hematological parameters were typically used to determine the effects of feed additives on fish health. Data obtained from our study showed that TPT exposure could reduce the amounts of Hct and Hb. Indeed, TPT has a wide range of toxicological effects as a contaminant [12], and it may have an inhibitory effect on the hematopoietic function of the fish, thus slowing down the rate of cell renewal in the hematopoietic system. However, the addition of SFN to the diet resulted in a remarkable increase in Hct amount, as well as an increase in RBC and Hb amounts, although no significant difference was observed. The increases in RBC, Hct, and Hb could facilitate tissue oxygenation and elimination of carbon dioxide [31]. The results of the present study were in agreement with the results of previous studies, indicating that the plant extracts positively affect hematological parameters in fish [32]. However, no studies in the literature had reported the effect of SFN on hematological parameters. In fish, there was a key role for the generation of innate immune components (e.g., complement and lysozyme) in the regulation of the immune response. C3, C4, and lysozyme were essential immune components with non-selective effects against bacteria and antigens. With the same tendency as lysozyme, ACP was a component of macrophage lysosomes. Fish immune systems, especially innate immunity, which changed, could be used as an early indicator of a response to oxidative stress due to its sensitivity to chemicals [33]. In this study, TPT exposure reduced the levels of C3, C4, ACH50, ACP, and LZM, that is, immunosuppression occurred. This was not surprising, since exposure to TPT for long term might cause chronic immunotoxic effects [34]. Similar results were reported in common carp (*Cyprinus carpio*) [12], zebrafish (*Danio rerio*) [35], and gastropod abalone (*Haliotis diversicolor*) [36]. On the other hand, the addition of SFN showed a significant increase in immunological parameters, including plasma C3 content and ACP and LZM activities, confirming that dietary intake of SFN markedly affected some crucial defense molecules in plasma [37] and effectively protected against TPT-induced immunotoxicity in *Cyprinus carpio haematopterus*. This result was in accordance with our previous study [18].

TNF-α, IL-1β, and IL-10 are often used as markers of inflammatory response. In this study, there was an increase in mRNA expression of *IL-1β* and *TNF-α* with TPT treatment, but a decrease in the mRNA expressions of the anti-inflammatory cytokine *IL-10* in the liver. This is further supported the fact that TPT gradually inhibited the occurrence of immune responses at the gene level [38]. COX2, as an important inflammatory mediator, is induced by a variety of stimuli, including cytokines. In our study, TPT exposure markedly increased the liver *COX2* mRNA expression; the result had the same trend as *TNF-α* and *IL-1β*. This result was expected, since the expression level of *COX2* in inflammatory cells increased rapidly and resulted in inflammatory responses and tissue damage when cells were stimulated by inflammation [39]. In addition, SFN supplementation efficiently alleviated the inflammatory response that was induced by TPT. This result confirmed the vital role of SFN in mediating inflammatory responses and immune function in animals [40]. Other studies on dietary plant extracts from *Cyprinus carpio* had observed similar results [41,42]. However, our previous study also had shown that the mRNA expressions of liver *IL-1β* and *TNF-α* both increased and then decreased with increasing dietary SFN levels from 0 to 20 mg kg^−1^ [18]. This might be explained by the ability of SFN to maintain the dynamic equilibrium of inflammatory factors in fish within a certain range of conditions.

Fish liver is correlated with nutrient deposition, biotransformation, metabolism, detoxification, and blood supply, and it was also one of the organs most affected by pollution. Changes in the liver may be useful as markers indicating prior exposure to environmental stressors. In this study, the liver of TPT-exposed fish illustrated hepatocyte hypertrophy, nuclear disappearance, hepatocyte vacuolization, and the dilation of hepatic sinusoids. Histopathological studies had shown that fish displayed compensatory reflexes such as dilation of hepatic sinusoids, hemorrhage, and intercellular vacuolization during exposure to toxicants [43]. Dilation of the hepatic sinusoids increased blood flow through tissue, which enabled speeding up the process of filtration to detoxify the fish [44]. Similarly, several studies had shown heavy metals in the environment could cause histopathological changes in the liver of fish [45,46]. On the contrary, the addition of SFN to the diet alleviated liver injury, with a reduced incidence of hepatocyte size, nuclear disappearance, and hepatocyte vacuolization with contraction of the hepatic sinusoids compared to the TPT group, which might be related to the function played by SFN. According to previous studies, SFN could improve antioxidant capacity and reduce immune stress in *Cyprinus carpio haematopterus* [18], while oxidative stress-associated molecular damages were eventually reflected in the impairments of the tissue structure [47].

Environmental pollution could lead to oxidative stress in biological systems. Oxidative stress was considered to play an essential role in many physiological and pathological phenomena in fish. In this study, TPT exposure resulted in a reduction in CAT, SOD, GPX activities, and GSH content, and an increase in MDA content in the liver of *Cyprinus carpio haematopterus*. Numerous studies have demonstrated the following: (1) GPX protected the organism mainly against the harmful effects of hydrogen peroxide; GSH is a widely available small molecule antioxidant found in cells, which could help the body to scavenge free radicals and peroxides [48]. (2) As the first line of defense against oxidative stress, the SOD-CAT system scavenged excess free radicals from the body and maintained a balance between the oxidative and antioxidant systems [18]. (3) MDA was a polyunsaturated fatty acid peroxidation product, mainly generated by free radicals, which could cause toxicity to the body and was a scientifically accepted oxidative stress indicator [49]. It also validated the fact that exposure to TPT could cause oxidative stress to the liver of fish, resulting in oxidative damage. The results were in line with previous studies on this species [50]. In contrast to TPT exposure alone, the incorporation of SFN into fish diets could increase CAT, SOD, GPX activities, and GSH content, but decrease MDA content. SFN was able to exert a protective effect because of its potential for antioxidant capacity; SFN reduced ROS production and enhances antioxidant enzyme activity in cells [51]. This might be attributed to the antioxidant property of sulfur in SFN, which could influence GSH metabolism, thus avoiding ROS accumulation and oxidative damage [52,53]. The results were in line with previous studies on *Cyprinus carpio haematopterus* [18,50], *Litopenaeus Vannamei* [15], and rats [54].

It has been shown that antioxidant parameters, such as SOD, CAT, and MDA in fish liver were positively related to their respective mRNA levels [55]. This was an important tool for investigating the effects of oxidative stress through gene mRNA expression. In this study, TPT exposure resulted in a remarkable decrease in hepatic *SOD* mRNA expression, but an increase in *MDA* mRNA expression of *Cyprinus carpio haematopterus*. We also found that TPT treatment decreased the mRNA expressions of liver *CAT* and *Nrf2*, although there was no significant difference. These genes confer protection against oxidative damage across various tissues, excluding *MDA*. This result was not surprising, since the reduced activity of antioxidant enzymes in TPT-treated fish might be partially attributed to the down-regulation of corresponding genes in liver tissue [9]. SFN supplementation partly prevented these alterations; this indicated that the addition of SFN was effective in restoring antioxidant indicator mRNA expression in TPT-exposed fish. According to previous studies, SFN could up-regulate the mRNA expression of *Nrf2* in fish, as might consequently boost antioxidant capacity [56]. Nrf2 plays an important key role in the elimination of reactive oxygen through the up-regulation of the expression of antioxidant-related genes (e.g., GPX) to resist oxidative stress [57]. This was supported by the fact that SFN was an activator of *Nrf2*, and SFN exerts a powerful antioxidant capacity in activating the Nrf2 signaling pathway [58]. Although SFN shows promising antioxidant and immune-enhancing effects in the present study, it is crucial to acknowledge that further research is necessary to evaluate its long-term effectiveness.

## 5. Conclusions

In conclusion, this study revealed the protective role of SFN against the physiological toxicity of TPT in *Cyprinus carpio haematopterus*. The results obtained in this study suggested that TPT exposure inhibited the growth, immune and antioxidant capacity, and altered the liver tissue structure. SFN could relieve these effects by its growth-promoting, immune-enhancing, and antioxidant capabilities. Based on these results, we proposed that SFN was an excellent aquaculture feed additive for reducing the negative effects of TPT contamination.

## Figures and Tables

**Table 1:** Diet composition of *Cyprinus carpio haematopterus*.

Ingredients (%)	
Fish meal	5.00
Soybean meal	32.00
Rapeseed meal	15.00
Cottonseed meal	15.00
Fish oil	1.00
Soybean oil	2.00
Wheat flour	21.00
Wheat bran	6.00
Ca(H_2_PO_4_)_2_	1.80
Premix ^1^	1.00
Salt	0.20
Proximate analyses (% air-dry basis)	
Dry matter	89.73
Crude lipid	4.04
Crude protein	35.82
Crude ash	9.45

^1^ Premixtures included vitamins (IU or mg kg^−1^) and minerals (g kg^−1^): V_A_, 900,000 IU; V_B1_, 320 mg; V_B2_, 1090 mg; V_B5_, 2000 mg; V_B6_, 500 mg; V_B12_, 1.6 mg; V_C_, 5000 mg; V_D_, 200,000 IU; V_E_, 4500 mg; V_K3_, 220 mg; Na_2_SeO_3_, 0.04 g; FeSO_4_·7H_2_O, 25 g; CuSO_4_·5H_2_O, 2.0 g; ZnSO_4_·7H_2_O, 22 g; MnSO_4_·4H_2_O, 7 g; CoCl_2_·6H_2_O, 0.1 g; KI, 0.026 g; choline, 60,000 mg; pantothenate, 1000 mg; folic acid, 165 mg.

**Table 2:** Sequence of primers for gene expression of *Cyprinus carpio haematopterus*.

Gene	Sequences of Primers (5′-3′)	Accession Numbers
*β-actin*	F: TTGACTTCCCTGACAGCTGT	M24113.1
	R: CGAATCCGGCTTTGCACATA	
*CAT*	F: ACAATGCAATCTCCATCGGC	XM_019068441.1
	R: CGTCTTGAGGTGCATTCTGG	
*SOD*	F: CACTGGCCTTACTCCTGGAA	JX977106.1
	R: GGTCCACCGTGAGTTTGATT	
*MDA*	F: GCTGCCAGTTGGGTGTTCTTTTA	DQ983598.1
	R: CCATCCGTGACTCCAGTTATCAT	
*TNF-α*	F: GCACCGAAACGACGGAAGA	AJ311800.2
	R: ACGACCATGTTTCCCCCAC	
*IL-10*	F: GGAGGGCTTTCCAGTGAGAC	MG759384.1
	R: TATCACACTTTGCCACCGCT	
*IL-1β*	F: CACCCGCTGGATTTGTCAGA	AB757756.1
	R: ATGGTTGAGGTGGTCAGTATGG	
*COX2*	F: TCCGGTATGTGCAAAGCCGG	JX807772.1
	R: CCGCAGATTTCAGAGCATTGTC	
*Nrf2*	F: CTCCCGAGACGAACAGAGAG	MG759384.1
	R: GGTGCTTGGACATCATCTCG	

*CAT*, catalase; *SOD*, superoxide dismutase; *MDA*, malonaldehyde; *TNF-α*, tumor necrosis factor α; *IL-10*, interleukin 10; *IL-1β*, interleukin-1β; *COX2*, Cyclooxygenase 2; *Nrf2*, nuclear factor E2 related factor 2.

**Table 3:** Effects of TPT and SFN on the growth performance of *Cyprinus carpio haematopterus*.

	Control	TPT	TPT + SFN	SFN	Two-Way ANOVA		
					TPT	SFN	Interaction
W_i_ (g)	56.97 ± 0.83	56.64 ± 0.87	57.48 ± 0.81	56.53 ± 1.00			
W_f_ (g)	110.47 ± 2.24 ^a^	98.04 ± 2.56 ^b^	112.26 ± 2.21 ^a^	111.28 ± 1.08 ^a^	*	**	*
WGR (%)	93.93 ± 1.16 ^a^	73.23 ± 5.35 ^b^	95.30 ± 2.30 ^a^	96.88 ± 2.07 ^a^	**	**	*
SGR (% day^−1^)	1.18 ± 0.04 ^a^	0.98 ± 0.05 ^b^	1.19 ± 0.02 ^a^	1.21 ± 0.02 ^a^	*	**	*
FCR	1.29 ± 0.07 ^b^	1.62 ± 0.08 ^a^	1.35 ± 0.02 ^b^	1.23 ± 0.07 ^b^	**	*	ns
CF (%)	2.31 ± 0.02 ^a^	1.95 ± 0.17 ^b^	2.32 ± 0.07 ^a^	2.47 ± 0.08 ^a^	*	*	ns
HSI (%)	1.49 ± 0.03 ^b^	1.91 ± 0.08 ^a^	1.67 ± 0.09 ^ab^	1.56 ± 0.04 ^b^	**	ns	*
VSI (%)	5.54 ± 0.24	5.79 ± 0.23	5.60 ± 0.26	5.44 ±0.15	ns	ns	ns

TPT, Triphenyltin; SFN, Sulforaphane; W_i_, the initial weight; W_f_, the final weight; WGR, Weight gain rate; FCR, Feed conversion ratio; SGR, Specific growth rate; CF, Condition Factor; HSI, Hepatosomatic index; VSI, Viscera index. Values are expressed means ± SEM. * *p* < 0.05, ** *p* < 0.01, ns: not significant. Values in the same line with different superscripts are significantly different (*p* < 0.05).

## Data Availability

Data will be made available on request.
